# *Corynebacterium accolens* inhibits *Staphylococcus aureus* induced mucosal barrier disruption

**DOI:** 10.3389/fmicb.2022.984741

**Published:** 2022-09-14

**Authors:** Shuman Huang, Karen Hon, Catherine Bennett, Hua Hu, Martha Menberu, Peter-John Wormald, Yulin Zhao, Sarah Vreugde, Sha Liu

**Affiliations:** ^1^Department of Surgery-Otolaryngology Head and Neck Surgery, Basil Hetzel Institute for Translational Health Research, Central Adelaide Local Health Network, Woodville South, SA, Australia; ^2^Adelaide Medical School, The University of Adelaide, Adelaide, SA, Australia; ^3^Department of Rhinology, The ENT Hospital, The First Affiliated Hospital of Zhengzhou University, Zhengzhou, China; ^4^Department of Otolaryngology, Head and Neck Surgery, Shanghai General Hospital, Shanghai Jiaotong University, Shanghai, China

**Keywords:** *Corynebacterium accolens*, *Staphylococcus aureus*, planktonic, biofilm, TER, cell-free culture supernatants

## Abstract

**Background:**

*Corynebacterium accolens* (*C. accolens*) is a common nasal colonizer, whereas *Staphylococcus aureus* (*S. aureus*) is typically regarded a pathogenic organism in patients with chronic rhinosinusitis (CRS). This study aims to evaluate the interaction of the two bacteria *in vitro*.

**Methods:**

Clinical isolates of *C. accolens* and *S. aureus* from sinonasal swabs, as well as primary human nasal epithelial cells (HNECs) cultured from cellular brushings of both healthy and CRS patients were used for this study. The cell-free culture supernatants of all isolates grown alone and in co-cultures were tested for their effects on transepithelial electrical resistance (TER), FITC-Dextran permeability, lactate dehydrogenase (LDH), and IL-6 and IL-8 secretion of HNECs. Confocal scanning laser microscopy and immunofluorescence were also used to visualize the apical junctional complexes. *C. accolens* cell-free culture supernatants were also tested for antimicrobial activity and growth on planktonic and biofilm *S. aureus* growth.

**Results:**

The cell-free culture supernatants of 3\*C. accolens* strains (at 60% for *S. aureus* reference strain and 30% concentration for *S. aureus* clinical strains) inhibited the growth of both the planktonic *S. aureus* reference and clinical strains significantly. The *C. accolens* cell-free culture supernatants caused no change in the TER or FITC-Dextran permeability of the HNEC-ALI cultures, while the cell-free culture supernatants of *S. aureus* strains had a detrimental effect. Cell-free culture supernatants of *C. accolens* co-cultured with both the clinical and reference strains of *S. aureus* delayed the *S. aureus*-dependent mucosal barrier damage in a dose-dependent manner.

**Conclusion:**

*Corynebacterium accolens* cell-free culture supernatants appear to inhibit the growth of the *S. aureus* planktonic bacteria, and may reduce the mucosal barrier damage caused by *S. aureus*.

## Introduction

The microbiota can be defined as ecological communities of commensal and pathogenic microorganisms found in and on all multicellular organisms (Belkaid and Hand, 2014). The microbial communities encode millions of genes and their associated functions, which work in tandem with human cells to maintain cellular homeostasis (Yatsunenko et al., 2012). A wealth of studies have established the microbiota as an important contributor to essential mammalian functions including metabolism (Trompette et al., 2014), serotonin biosynthesis (Lynch and Pedersen, 2016), neurotransmission (Cryan and O'Mahony, 2011; Surjyadipta and Lukiw Walter, 2013), and immunomodulation (Marcus and Hornef Mathias, 2014; Lloyd Clare and Marsland Benjamin, 2017). The host-microbiota interface is particularly important with evidence suggesting that many chronic inflammatory diseases are associated with significant shifts in the local microbiota towards inflammatory configurations (Hand Timothy et al., 2016). Therefore, a better understanding of the microbiota associated with these conditions may be the key to elucidating their underlying pathogenesis and ultimately facilitating the development of new treatments.

Chronic rhinosinusitis (CRS) is a persistent inflammatory condition of the paranasal sinus mucosa. Although its pathogenesis remains unclear, it is believed that external factors such as fungi, superantigens and toxins produced by bacteria and bacterial biofilms, as well as mucosal barrier disruption and a dysregulated innate immune response play a role (Jiao et al., 2019; Blot et al., 2020). Sinonasal microbiota studies have shown that healthy people and CRS patients have a similar overall bacterial burden and share many common phyla (Psaltis and Wormald, 2017), however patients with CRS tend to have reduced bacterial diversity with an expansion of opportunistically pathogenic microorganisms, such as *Staphylococcus aureus*, *Staphylococcus epidermidis*, and *Propionibacterium acnes*. Similar to other chronic inflammatory conditions, it is possible that pathogen expansion and the microbiota imbalance may be an initial cause of the chronic immune response and inflammation seen in this condition (Hand Timothy et al., 2016).

Our department’s previous research characterised the sinonasal microbiota and its global geographical variations in health and CRS using a large international patient cohort. Using 16S rRNA amplicon sequencing, we determined that *Corynebacterium* and *Staphylococcus* were amongst the most dominant genera in the majority of patients, regardless of their disease state. Unfortunately it was not possible to accurately characterize the bacteria genus to the species level due to the well-documented limitations of the short-read gene sequencing, where sequencing the V3-V4 hypervariable region of the 16S rRNA gene and taxonomy assignment was perfomed against the Greengenes 16S reference database. Several different *Corynebacterium* species are known to inhabit the nasal cavity, with the majority believed to be potential mutualists with important protective functions. *Corynebacterium accolens* (*C. accolens*), for example, is a common nasal colonizer and can inhibit *Streptococcal* growth *via* the release of oleic acids from the hydrolysis of host triacylglycerols (Bomar et al., 2016). Conversely, *Staphylococcus aureus* (*S. aureus*) is considered to be pathogenic in patients with CRS with its presence associated with recalcitrant CRS and poorer postoperative outcomes (Archer et al., 2011; Singhal et al., 2011; Cleland et al., 2014).

Previous research from our department has shown that *C. accolens,* when isolated from a healthy human nasal cavity, can exhibit antimicrobial activity against planktonic and biofilm growth of *S. aureus* and methicillin-resistant *S. aureus* (MRSA) isolated from CRS patients (Menberu et al., 2021b). This study aims to build on previous research by evaluating the interactions between the clinical isolates of *C. accolens* and *S. aureus,* cultured from the sinonasal mucosa, with a particular focus on their effects on the mucosal barrier in an *in vitro* setting.

## Materials and methods

The study was approved by the Central Adelaide Local Health Network Human Research Ethics Committee (HREC/15/TQEH/132) and written informed consent was obtained from participants before collection of microbial swabs and primary human nasal epithelial cells (HNECs).

## Sample collection

Bacterial swabs were used to sample the middle meatus of CRS patients and non-CRS controls. Cytobrushes (EndoScan Brush, Medico, Melbourne, VIC, Australia) were used intraoperatively to harvest primary human nasal epithelial cells (HNECs) from the inferior turbinate mucosa. Control patients were patients undergoing endoscopic skull base procedures without clinical or radiological evidence of sinus disease. CRS patients fulfilled the diagnostic criteria set out in the position papers by the American Academy of Otolaryngology and Head and Neck Surgery and the European Position Statement (EPOS) on CRS (Fokkens et al., 2012; Lin and Nnacheta, 2015).

## Bacterial assay

### Bacterial culture

To understand if *C. accolens* cell-free culture supernatants can inhibit *S. aureus* planktonic growth, three *C. accolens* clinical isolate strains (C1 to C3) were selected from our bacterial biobank, based on their established broad antagonistic activity against multiple methicillin-resistant *Staphylococcus aureus* (MRSA) and methicillin-susceptible *Staphylococcus aureus* (MSSA) clinical isolates in our previously published work by Menberu et al. (2021a,b); *S. aureus* clinical isolates (SC) were identified by South Australia (SA) Pathology and *S. aureus* reference strain (ATCC51650, SA) was purchased from American Type Culture Collection (ATCC, Manassas, United States). *S. aureus* strains were frozen in tryptone soy broth (TSB, Thebarton, SA, Australia) with 20% (v/v) glycerol at-80°C until use. Isolates were thawn and cultured at 37°C for 24 h on 1.5% trypticase soy agar (TSA) plates with 0.5% Tween 80 (Sigma-Aldrich, St. Louis, United States). For each of the *C. accolens* and *S. aureus* isolates, a 0.5 MacFarland Unit (MFU) suspension was created in 0.9% sodium chloride (NaCl). The suspension was subsequently diluted 1: 100 in 10 ml TSB supplied with 0.5% Tween 80 and incubated at 37°C on an orbital shaking incubator at 180 rpm for 24 h. The overnight bacterial culture was then diluted with TSB containing 0.5% Tween 80 to an absorbance of 0.05 at a wavelength of 600 nm (OD_600_) (SmartSpec 3000, Biorad, CA, United States). 10 ml of the diluted bacterial suspension was transferred into a 100 ml centrifuge tube and incubated at 37°C on an orbital shaking incubator at 180 rpm. The OD_600_ was measured hourly to prepare a standard growth curve of the bacteria.

### Cell-free culture supernatants harvest and protein quantification

For planktonic cell-free culture supernatants, 0.5 McF *C. accolens* and *S. aureus* suspension in 0.9% NaCl were obtained using a single colony from a plate grown on 1.5% TSA with 0.5% Tween 80 at 37°C for 24 h. The bacterial suspension was diluted at 1: 100 in 10 ml TSB with 0.5% tween 80 in a 50 ml falcon tube, then, the suspension of *C. accolens* or *S. aureus* was incubated at 180 rpm in a 37°C incubator in air for 24 h. For *C. accolens* and *S. aureus* co-cultures, a fixed number of *S. aureus* (5 × 10^5^ CFU) with *C. accolens* in different ratios ((50% (2.5 × 10^5^ CFU),70% (3.5 × 10^5^ CFU), and 90% (4.5 × 10^5^ CFU)) was incubated at 180 rpm in a 37°C incubator in air for 24 h.

After 24 h incubation, the cell-free culture supernatants from single cultures and co-cultures was spun down and filtered through a 0.22 μm syringe filter (Pall Corporation, San Diego, United States).

For biofilm cell-free culture supernatants, 1 McF *C. accolens* and *S. aureus* suspension was diluted 1: 15 in TSB with 0.5% tween 80 to form biofilm in 6-well plates (2 ml per well) (Fokkens et al., 2012). The suspension of *C. accolens*, *S. aureus,* and S*. aureus* co-cultured with *C. accolens* in different ratios (50% (2.5 × 10^5^ CFU),70% (3.5 × 10^5^ CFU), and 90% (4.5 × 10^5^ CFU)) was incubated for 48 h at 37°C on a gyratory shaker at 70 rpm in air. The cell-free culture supernatants were harvested as described above.

Then the protein concentration was determined using Nano Orange protein quantitation kit (Invitrogen, Carlsbad, CA, United States). The experiment was repeated three times.

## Antibacterial assay

### Planktonic bacteria assay

100 μl of *S. aureus* suspension (0.5 McF) was grown in TSB with 0.5% Tween 80 in a 96-well plate (Corning Incorporated, Corning NY, United States) containing different concentrations (20–90% v/v) of *C. accolens* (clinical isolates 1 to 3) cell-free culture supernatants and incubated for 24 h at 180 rpm in 37°C in air. *S. aureus* treated with TSB with 0.5% Tween 80 was used as positive control and TSB with 0.5% Tween 80 without *S. aureus* as a negative control. The OD_600_ was measured to determine the growth of bacteria after 24 h treatment. All treatments were carried out in six replicates and the entire experimental procedure was repeated three times.

### Confocal laser scanning microscopy

Confocal laser scanning microscopy was used to confirm inhibition of planktonic cells (Lin and Nnacheta, 2015). Briefly, one drop of cells from above was spotted on the glass slide and left to air dry. Cells were then stained with a LIVE/DEAD BacLight Bacterial Viability Kit (Life Technologies Australia, Mulgrave, Victoria, Australia) according to the manufacturer’s instructions. The stained cells were examined at 20× magnification using a confocal laser scanning microscope (Zeiss LSM700, Carl Zeiss AG, Oberkochen, Germany). The experiment was repeated three times.

### Biofilm assay

Black 96-well microplates (Costar; Corning Incorporated, Corning, NY, United States) were used to form biofilms. In Brief, a 1.0 McF *S. aureus* suspension in 0.9% NaCl was diluted 1: 15 in TSB and gently mixed by inversion. 150 μl/well of the resulting suspension was plated into a 96-well microplates in six replicates. The top and bottom edge of the plate were filled with 200 μl of sterile phosphate-buffered saline (PBS) to prevent dehydration. Negative controls were added to each plate containing TSB solution only. The plates were then incubated for 48 h at 37°C on a gyratory shaker at 70 rpm to allow for biofilm formation. After 48 h, biofilms were rinsed three times with 1 × PBS to remove planktonic bacteria. Following that, the *S. aureus* biofilm was treated with *C. accolens* planktonic and biofilm cell-free culture supernatants (in 20–100% v/v) for 24 h. *S. aureus* biofilms treated with TSB only were included as a positive control. After treatment, the metabolic activities of biofilm were determined using an alamarBlue assay (Sigma-Aldrich, St. Louis, MO, United States; Menberu et al., 2021b). Briefly, the microplates were incubated with 200 μl diluted (1: 10 ratio) alamarBlue and incubated at 37°C for 3 h. Subsequently, the fluorescence intensity of the samples was measured using a FLUOstar Optima 96-well fluorescence microplate reader (BMG Labtech, Ortenberg, Germany) at λexcitation = 530 nm/λemission = 590 nm. All assays were carried out in six replicates and the experiment was repeated three times.

## Cell culture assays with primary human nasal epithelial cells

### Primary human nasal epithelial cells

HNECs were cultured as previously described (Murphy et al., 2018a; Ramezanpour et al., 2018). Briefly, HNECs were suspended in 10 ml DMEM medium (Gibco, Thermofisher Scientific, Melbourne, VIC, Australia) and centrifuged at 300× *g* for 5 min at 4°C. The pellet was then resuspended in 1 ml PneumaCult™-Ex Plus Basal Medium (STEMCELL Technologies, Tullamarine, VIC, Australia) and plated on a 100-mm diameter culture plate coated with anti-CD68 antibodies (Dako, Glostrup, Denmark) for 20 min at 37°C to deplete monocytes. Then HNECs were seeded in collagen-coated T75 cell culture flasks (Corning Incorporated, NY, United States) and grown in 15 ml Ex-medium consisting of PneumaCult™-Ex Plus Basal Medium (STEMCELL Technologies, Tullamarine, VIC, Australia), PneumaCult™-Ex Plus 50× Supplement (STEMCELL Technologies, Tullamarine, VIC, Australia), and penicillin–streptomycin (Thermo Scientific, Walthman, MA, United States). The seeded HNECs were incubated at 37°C with a 95% humidity incubator supplied with 5% CO_2_ and inspected daily under light microscopy.

### Air liquid interface culture

Once HNECs reached 80–100% confluence, cells were detached by treating them with 0.05% trypsin (Thermo Scientific, Waltham, MA, United States) and resuspended in Ex-medium (PneumaCult™-Ex Plus Basal Medium; PneumaCult™-Ex Plus 50× Supplement and penicillin–streptomycin prepared as above description) after centrifugation. Then, cell suspensions were plated onto the apical collagen IV-coated chambers of Transwells (BD Biosciences, San Jose, California, United States). 500 μl Ex-medium was added in the basolateral chamber. Cells were given 2 days to settle and the medium from the apical chamber was removed completely and the basolateral chamber medium was changed to PneumaCult™-ALI Basal Medium (STEMCELL Technologies, Tullamarine, VIC, Australia); PneumaCult™-ALI 10× Supplement; penicillin–streptomycin/amphotericin B (Thermo Scientific, Walthman, MA, United States); And PneumaCult™-ALI Maintenance Supplement (STEMCELL, Vancouver, Canada). The basolateral chamber medium was replaced every 3 days. The cells were cultured for a period of 17 up to 21 days.

### Transepithelial electrical resistance

TER was measured by using an EVOM volt-ohmmeter (World Precision Instruments, Sarasota, FL, United States). Briefly, 100 μl of PneumaCult™-ALI Basal Medium was added to the apical chamber of ALI cultures to form an electrical circuit across the cell monolayer and into the basal chamber. Cultures were maintained at 37°C during the measurement period using a heating platform (LEC Instrument, Australia). Only wells displaying baseline resistance readings greater than 700 Ω/cm^2^ were used for the experiments. *S. aureus* and *C. accolens* co-cultured cell-free culture supernatants in different ratios were added to the apical chambers of each Transwell. TER measurements were taken at times 0, 0.5, 1, and 2 h. As the negative and postitive controls, ALI cultures treated with TSB and 2% Triton X-100 (Promega, Madison, WI, United States) were tested together. The experiment was repeated three times.

### FITC-dextran permeability assay

Paracellular permeability was assessed by measuring the apical-to-basolateral flux of FITC-Dextran 4 kDa (Sigma, Saint Louis, United States). Briefly, after treating cells with bacterial cell-free culture supernatants and TSB (negative control) and 2% Triton X-100 (positive control) for 2 h, the apical chambers were filled with 3 mg/ml of FITC-Dextran and incubated at 37°C for 2 h. Samples were then taken from the basolateral compartment and transferred to a clear bottom black 96-well plate (Corning-Costar Corp., Cambridge, United Kingdom). The fluorescence of the samples was measured with a FLUOstar Optima 96-well fluorescence microplate reader (BMG Labtech, Ortenberg, Germany) at excitation and emission wavelengths of 485 nm and 520 nm. The experiment was repeated three times.

### Immunofluorescence staining

Following the FITC-Dextran Permeability Assay, cells were fixed for 10 min on ice with 2.5% formalin in PBS. Fixed samples were then permeabilized with 1% sodium dodecyl sulfate (SDS) for 10 min on ice. Following this, the permeabilized cells were blocked for 1  h with serum-free blocker (Dako, Glostrup, Denmark) at room temperature. Cells were incubated with the primary antibody [Rabbit anti-claudin-1 (1:50; Invitrogen, Carlsbad, CA, United States); Mouse anti-zonula occludens-1 (1,100; Invitrogen, Carlsbad, CA, United States)] overnight at 4°C. Following washing, cells were then incubated with secondary antibodies [Donkey anti-rabbit Cy3 (1:200; Jackson ImmunoResearch Labs Inc., West Grove, PA, United States); Donkey anti-mouse IgG Alexa Fluor 488 (1:200; Jackson ImmunoResearch Labs Inc., West Grove, PA, United States)] for 1 h at room temperature (RT) and with 200 ng/ml of 4′,6-diamidino-2-phenylindole (DAPI; Sigma-Aldrich, St. Louis, MO, United States) for 10 min at RT to resolve nuclei. Cells were then mounted with a fluorescence anti-fade mounting medium (Dako, Glostrup, Denmark) and were covered with coverslips. Images were examined with a confocal laser-scanning microscope LSM700 (Zeiss Microscopy, Jena, Germany) and images were then processed with ZEN Imaging Software (Carl Zeiss AG, Oberkochen, Germany). The experiment was performed in triplicates.

### Cell cytotoxicity assay

Following the last TER measurements (2 h), the medium from the basal chambers of each sample was collected and cytotoxicity was determined using the lactate dehydrogenase (LDH) release kit (Promega, Madison, WI, United States) according to the manufacturer’s instructions (Dong et al., 2020). Briefly, 50 μl of the medium from each condition was transferred to a new plate, and 50 μl of LDH reagent was added to the medium and this was incubated for 30 min in the dark at RT. Absorbance was read using a microplate reader at 490 nm (BMG Labtech, Ortenberg, Germany). Cells treated with TSB and 2% Triton X-100 were used as negative and positive control, respectively. The relative viability was calculated relative to the LDH levels of negative controls and positive controls. The experiment was performed in triplicates.

### Enzyme-linked immunosorbent assay

The medium was collected from the basolateral compartment of treated HNEC-ALI cultures after exposure with bacterial cell-free culture supernatants. Interleukin-6 (IL-6) and Interleukin-8 (IL-8) levels were estimated with an ELISA kit (BD Biosciences, New Jersey, United States), according to the manufacturer’s instructions. All measurements were performed in triplicates using a FLUOstar OPTIMA plate reader (BMG Labtech, Ortenberg, Germany). The IL-6 and IL-8 concentration was calculated from a standard curve and corrected for protein concentration.

## Statistical analysis

GraphPad Prism 9.0 (San Diego, CA, United States) was used for statistical analysis. One-way analysis of variance (ANOVA) followed by Dunnett’s multiple comparisons test or Tukey’s multiple comparisons test was used to compare the differences between multiple groups. A *p* < 0.05 was considered as statistically significant.

## Results

### Antibacterial effects of *Corynebacterium accolens* cell-free culture supernatants against planktonic and biofilm *Staphylococcus aureus*

**Figure 1:**
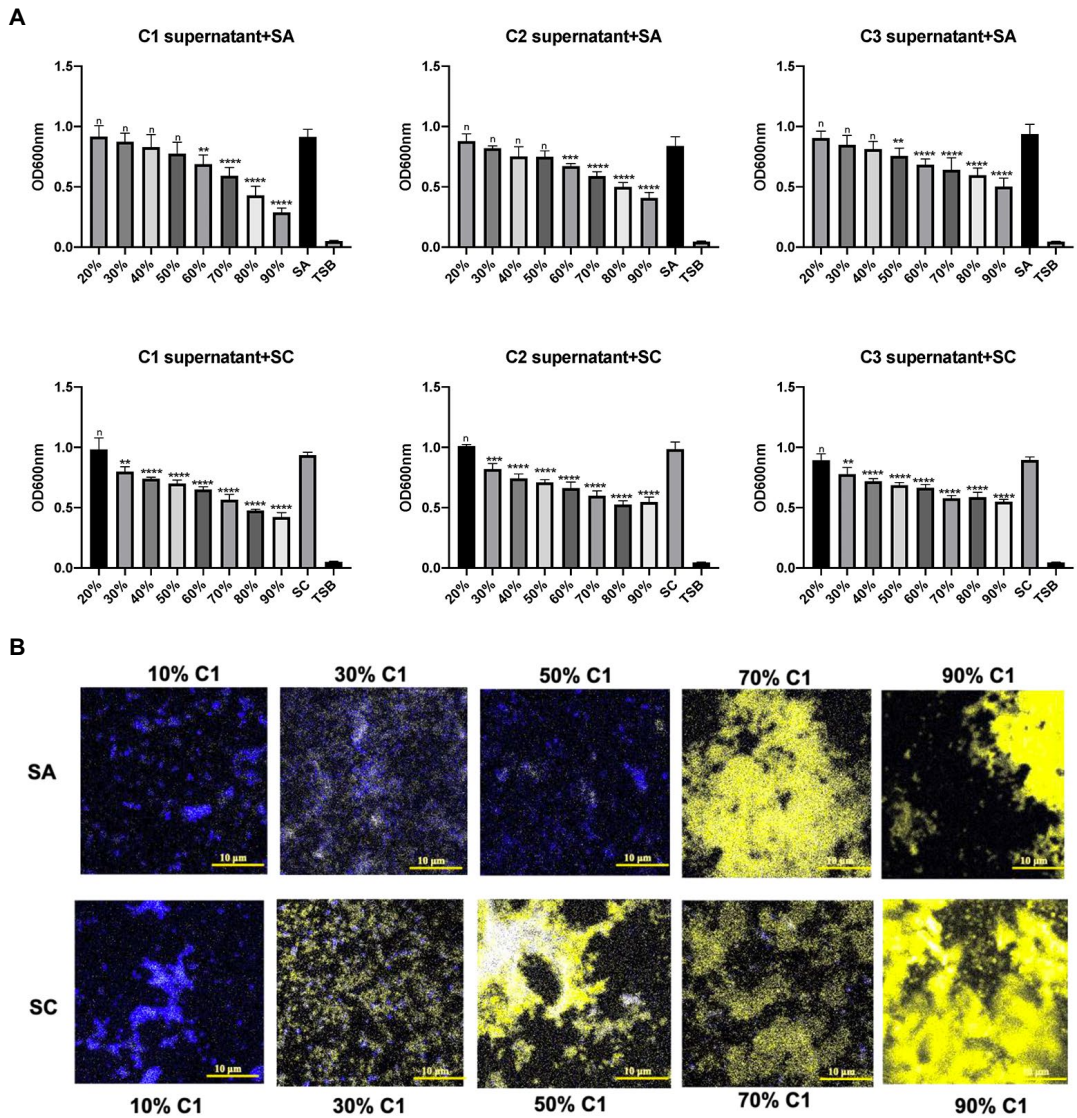
Antibacterial effects of *Corynebacterium accolens* cell-free culture supernatants against planktonic *S. aurens.* Different concentrations of cell-free culture supernatants from 3 clinical isolates of *C. accolens* were added to treat planktonic SA and SC. **(A)** OD_600_ was measured to determine the bacterial growth after 24 h treatment. **(B)** LIVE/DEAD BcalLight Bacterial Viability staining of *S aureus* planktonic bacteria was used to show dead (PI, yellow) and live (SYTO^®^ 9, blue) in samples representatives of each treatment group. C1 supernatant, *C. accolens* cell-free culture supernatants from *C. accolens* clinical isolate 1. C2 supernatant, *C. accolens* cell-free culture supernatants from *C. accolens* clinical isolate 2. C3 supernatant, *C. accolens* cell-free culture supernatants from *C. accolens* clinical isolate 3. SA, *S. aureas* ATCC 51650. SC, *S. aureas* clinical strain. Significance was determined by comparing the OD_600_ value of each treatment group with SA or SC untreated group. **p < 0.001, ***p < 0.001, ****p < 0.0001. n, no significant difference. Experiments were performed three times.

Three *C. accolens* clinical isolates (C1–C3) demonstrated broad antagonistic activity against a variety of methicillin-resistant *Staphylococcus aureus* (MRSA) and methicillin-susceptible *Staphylococcus aureus* (MSSA) clinical isolates chosen for this study. The cell-free culture supernatants of all three tested *C. accolens* were found to inhibit the growth of planktonic *S. aureus* in a dose-dependent way. There was significant growth inhibition of the reference strain at >50% v/v and of the *S. aureus* clinical strain at 30% v/v (Figure 1A). This was further confirmed using LIVE/DEAD BacLight Bacterial Viability staining (Figure 1B). Biofilm growth of both the reference and clinical *S. aureus* strains was not inhibited by the same concentrations of *C. accolens* cell-free culture supernatants (Supplementary Figure 1).

### The co-culture of *C. accolens* and *S. aureus* has no effect on measured total exoprotein secretion

To determine whether the presence of *C. accolens* live bacteria inhibited the growth of *S. aureus* and *S. aureus* exoprotein secretion, *S. aureus* clinical isolates and *S. aureus* ATCC51650 were co-cultured with various numbers of *C. accolens* live bacteria. Both planktonic and biofilm cell-free culture supernatants were harvested and proteins quantified. According to our finding, there was no significant difference for both planktonic and biofilm’ total exoprotein concentrations among all the experimental conditions we have tested (Supplementary Figures 2A,B).

## Effect of *Corynebacterium accolens* on *Staphylococcus aureus* induced HNECs barrier disruption

### *Corynebacterium accolens* cell-free culture supernatants reduce *Staphylococcus aureus* cell-free culture supernatants-induced transepithelial electrical resistance reduction

**Figure 2:**
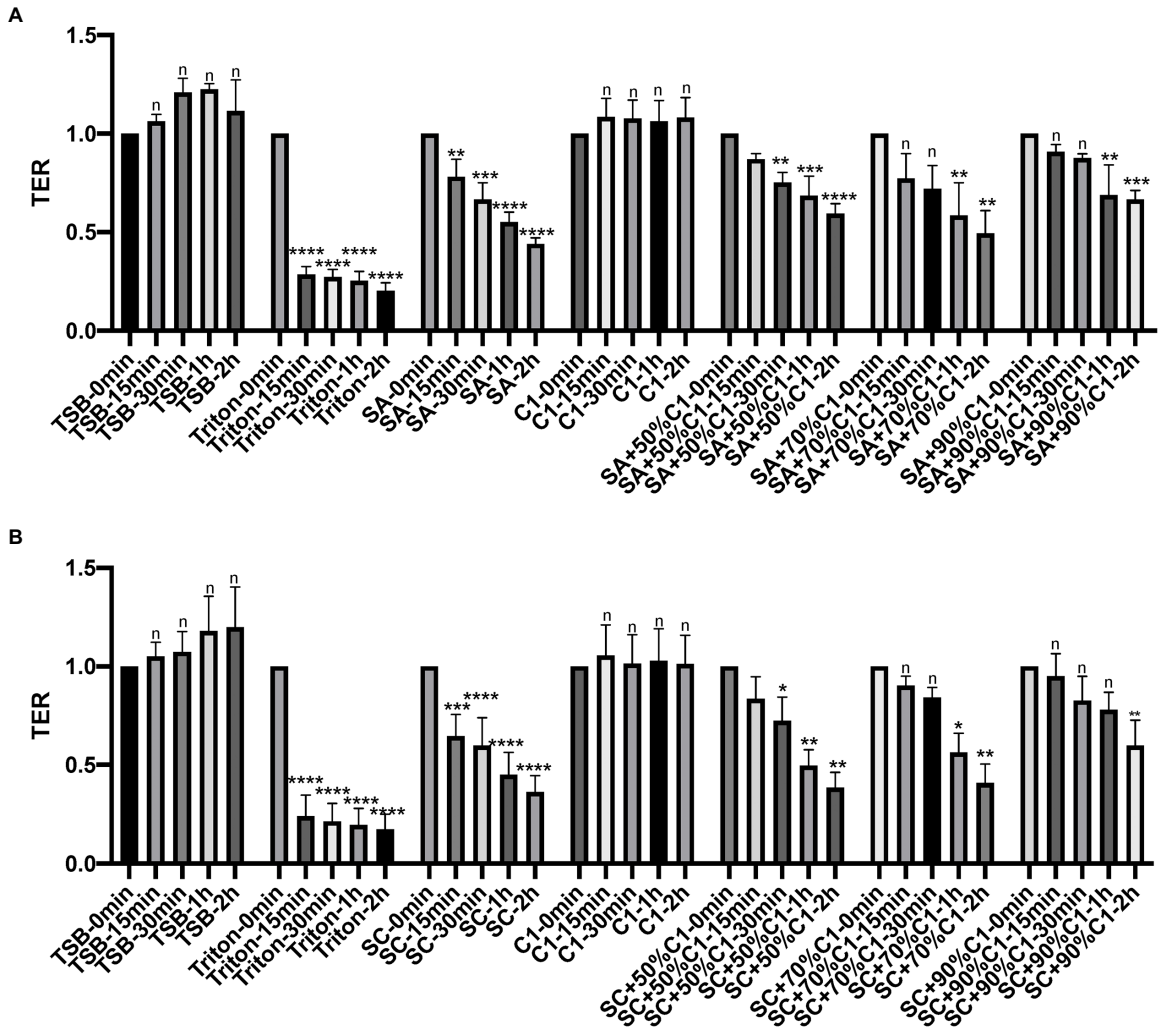
*Corynebacterium accolens* cell-free culture supernatants delay with *S. aureus* cell-free culture supernatants induced Transepithelial electrical resistance (TER) reduction. TER of HNEC-ALI was measured at 0 mins, 15 mins, 30 mins, 1 h and 2 h, following exposure to *C. accolens* cell-free culture supernatants, cell-free culture supernatants from the co-cultured *C. accolens*-*S. aureus* in different ratio and cell-free culture supernatants of *S. aureus* ATCC strain [(SA) **A**] or a clinical isolate of *S. aureus* [(SC) **B**]. TSB served as the negative control and Triton treatment as the positive control. Experiments were performed in triplicates. The significance was determined by comparing time point at 15 mins, 30 mins, 1 h and 2 h with time 0 min. *p < 0.05, **p < 0.01, ***p < 0.001, ****p < 0.0001. n, no significance difference.

To examine the effect of *C. accolens* cell-free culture supernatants on the HNECs barrier, HNECs were harvested from three control patients and three chronic rhinosinusitis without Nasal Polyps (CRSsNP) patients. TER reduction was then used to determine the effect of bacterial cell-free culture supernatants on the integrity and transcellular permeability of HNEC-ALI cultures. In our previous data (Menberu et al., 2021b), 3\*C. accolens* strains (C1–C3) exhibited comparable antimicrobial activities against two *S. aureus* strains. Consequently, one representative *C. accolens* (C1) strain was chosen for the remainder of these experiments. C1 cell-free culture supernatants did not affect the TER at any of the exposure times, while the reference (SA) and clinical strains of *S. aureus* (SC) cell-free culture supernatants reduced the TER significantly within 15 min and at all other time points (30 min, 1 and 2 h). Cell-free culture supernatants from the mixed *C. accolens-S. aureus* cultures induced a time and dose-dependent reduction in TER. With a higher concentration of *C. accolens* (70 and 90%), a statistically significant reduction in TER took 1 h to occur, while at lower numbers of *C. accolens* (50%), a significant reduction in TER occurred within 30 min (Figures 2A,B).

### *Corynebacterium accolens* cell-free culture supernatants reduce *Staphylococcus aureus* cell-free culture supernatants-induced detrimental effects on the HNEC-ALI cultures paracellular permeability

**Figure 3:**
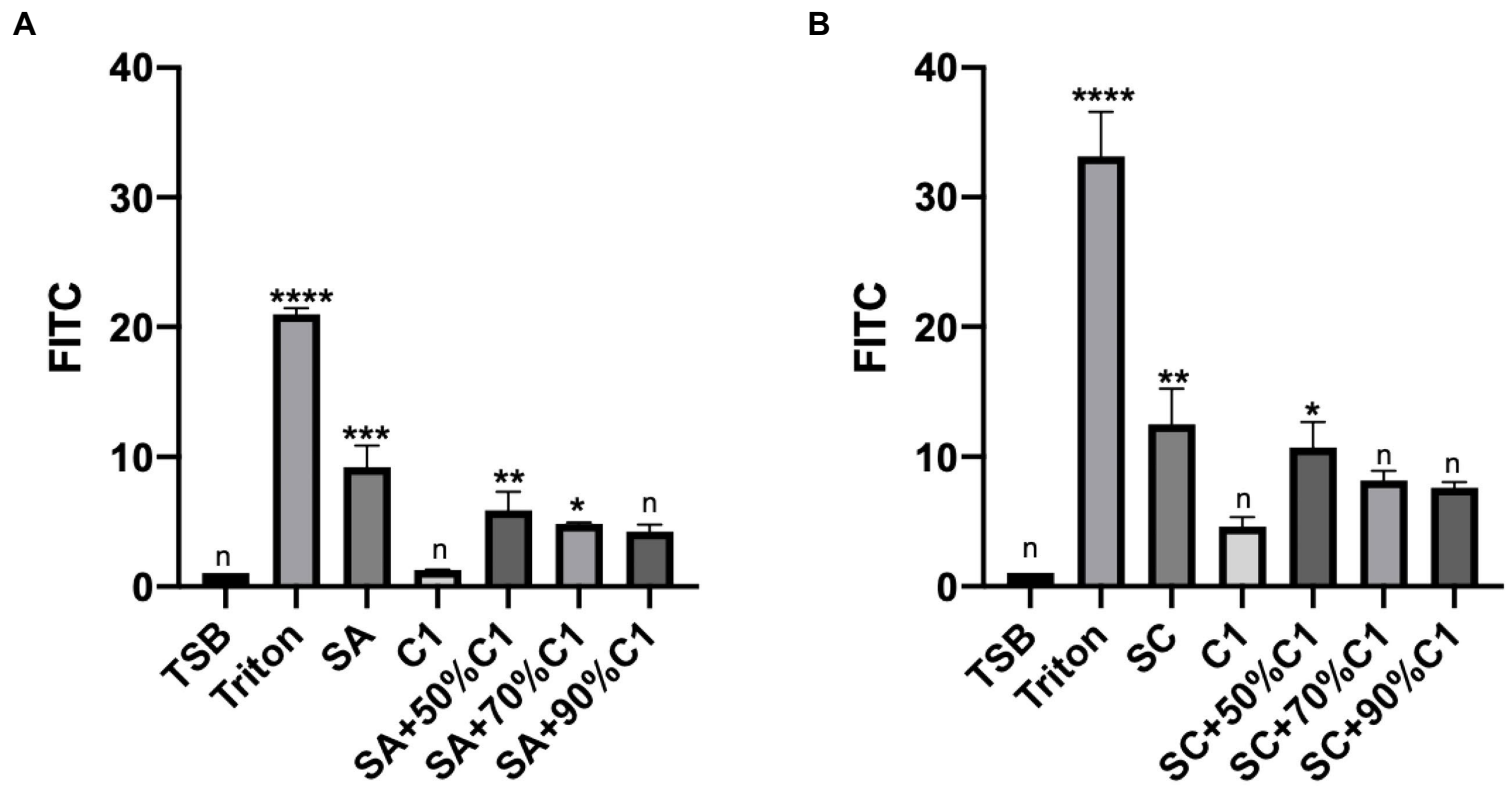
*Corynebacterium accolens* cell-free culture supernatants reduced with HNEC-ALI cultures paracellular permeability. The paracellular permeability.as measured by the FITC-Dextran Assay at 120 mins, following exposure to *C. accolens* cell-free culture supernatants, *C. accolens* cell-free culture supernatants from the mixed *C. accolens*-*S aureus* in different ratio and *S. aureus* ATCC strain [(SA) **A**] or a clinical isolate of *S. aureus* [(SC) **B**]. TSB served as the negative control and Triton treatment as the positive control. The significance was determined by comparing different treatment group with the negative control (TSB). Experiments were performed in triplicates. *p < 0.05, **p < 0.01, ***p<0.001, ****p < 0.0001. n, no significance difference.

To determine the effect of bacterial cell-free culture supernatants on the paracellular permeability of the epithelial cell layer, HNEC-ALI cultures were exposed for 2 h to the cell-free culture supernatants of *C. accolens* C1 and the reference and clinical strains of *S. aureus* co-cultured cell-free culture supernatants. The cell-free culture supernatants of *C. accolens* C1 appeared to have no significant effect on the paracellular permeability of the HNEC-ALI cultures when applied alone. Both the reference and clinical strains of *S. aureus* significantly increased the permeability of the epithelial cell layer. When the co-cultured *S. aureus* and *C. accolens* cell-free culture supernatants were applied, it was found that a higher ratio of *C. accolens* (90%) resulted in a greater reduction of *S. aureus*-dependent effects on paracellular permeability, compared to when a lower ratio (50%) of *C. accolens* was applied (Figures 3A,B).

### *Corynebacterium accolens* cell-free culture supernatants reduce *Staphylococcus aureus* cell-free supernatants-induced detrimental effects on HNEC-ALI cultures tight junctions

**Figure 4:**
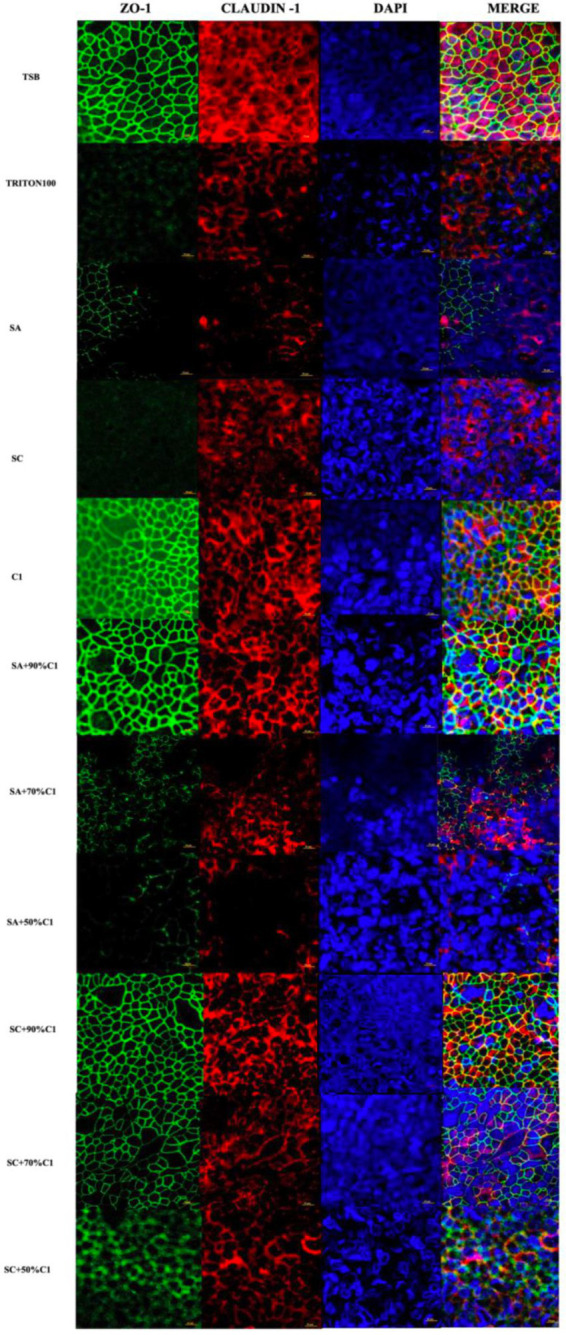
*Corynebacterium accolens* cell-free culture supernatants reduce *S. aureus* cell-free culture supernatants-induced detrimental effects on HNEC-ALI cultures tight junctions. Immunofluorescence staining of tight junction proteins of HNEC-ALI cultures treated with cell-free culture supernatants from SA and SC co-cultured with *C. accolens* in different ratios. HNEC-ALI cultured cells were stained with antibodies against Z0-1(green), claudin-1 (red) and DAPI to resolve nuclei (blue). TSB treatment was used as the negative control. Triton-100 was used as the positive control. Images were examined with confocal laser-scanning microscope (Scale bar=10 μm). C1, *C. accolens* clinical isolate 1; SA, *S. aureus* ATCC51650; SC, *S. aureus* clinical strain.

Zonula Occludens-1 (ZO-1) and Claudin-1 immunofluorescence were assessed on HNEC-ALI cultures following the application of the various bacterial co-cultured cell-free culture supernatants to determine the effect of these supernatants on the tight junction protein production and immunolocalization. *C. accolens* cell-free culture supernatants were found to have no significant effect on either of the tight junction proteins compared with control (TSB; Figure 4). Conversely, the cell-free culture supernatants from both the reference and clinical strains of *S. aureus* reduced the ZO-1 and Claudin-1 protein expression. In addition, as the concentration of live *C. accolens* bacteria increased during the co-cultivation with the *S. aureus*, the effect of their co-cultured cell-free culture supernatants on both tight junction proteins diminished.

### *Corynebacterium accolens* cell-free culture supernatants are not cytotoxic To HNEC-ALI cultures

An LDH assay was used to determine if all of the aforementioned changes were due to cytotoxicity. The cell-free culture supernatants from individual bacteria or co-cultured bacteria in all tested ratios exhibited no significant cytotoxicity when applied to HNEC-ALI cultures for 2 h (Supplementary Figures 3A,B).

### *Corynebacterium accolens* cell-free culture supernatants reduced *Staphylococcus aureus* cell-free culture supernatants-induced inflammatory cytokine production to baseline

**Figure 5:**
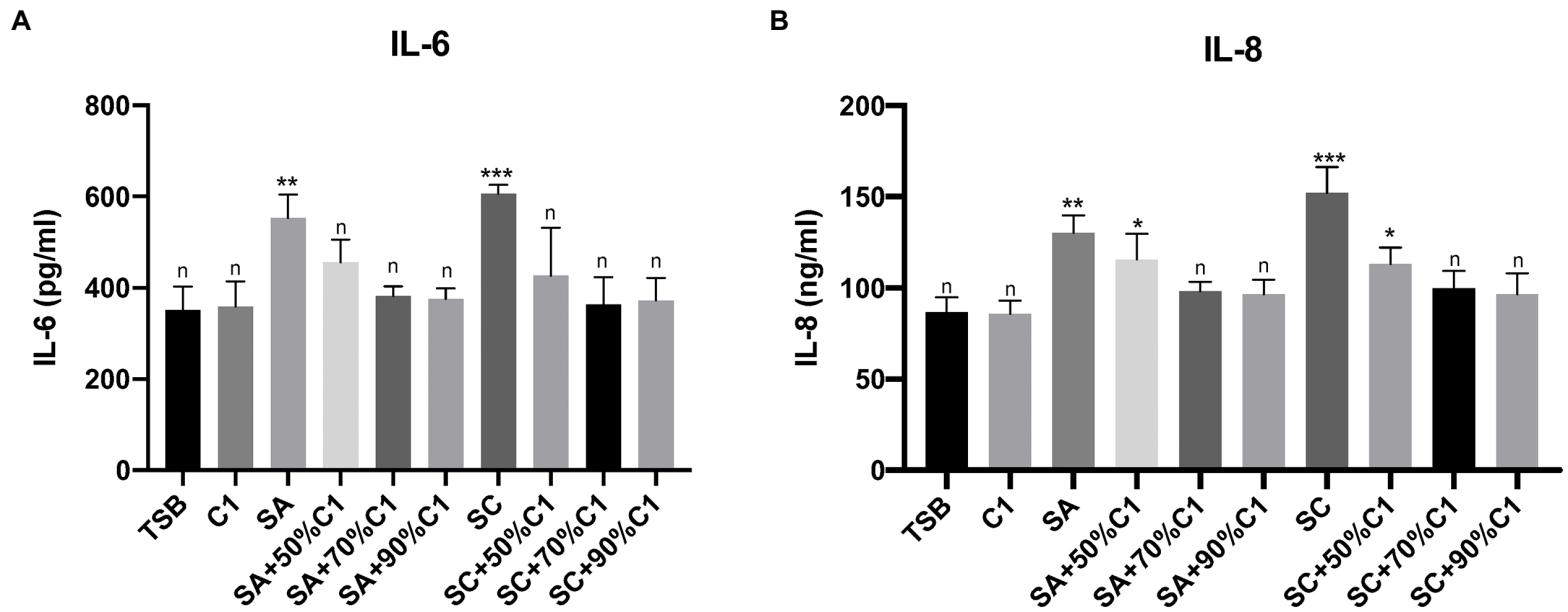
*Corynebacterium accolens* cell-free culture supernatants reduce *S. aureus* cell-free culture supernatants-induced inflammatory cytokine production to baseline. The IL-6 **(A)** and IL-8 **(B)** release by the HNEC-ALI cultures treated with the cell-free culture supernatants of *C. accolens*, *S. aureus and* co-cultured *C. accolens*, *S. aureus* in different ratio was measured. C1, *C. accolens* clinical isolate 1. SA, *S. aureus* ATCC51650. SC, *S. aureus* clinical strain. Experiments were performed in triplicates. *p < 0.05, **p < 0.01, ***p < 0.001. n, no significance difference. Experiments were performed three times.

Using ELISA to quantify IL-6 and IL-8 release from HNEC-ALI cultures after exposure to *C. accolens* C1 cell-free culture supernatants, we did not observe a significant increase in either IL-6 or IL-8 secretion. A significant increase was observed with exposure to both the reference and clinical strains of *S. aureus* cell-free culture supernatants after 2 h treatment; lower rates of IL-6 secretion were observed when HNEC-ALI cultures were exposed to the cell-free culture supernatants of *C. accolens* C1 co-cultured with *S. aureus*, in comparison to the control (TSB) and *C. accolens* C1 cell-free culture supernatants treatment. Cell-free culture supernatants that were harvested from the lower ratio of *C. accolens* C1 (50%) co-cultured with *S. aureus* did not reduce the IL-8 release, compared to control. However, when the ratio of *C. accolens* C1 increased (70 and 90%), the co-cultured cell-free culture supernatants reduced the release of IL-8 back to baseline levels (Figures 5A,B).

## Discussion

*Corynebacterium accolens* is typically regarded as a commensal bacteria in the sinuses of healthy patients. It is thought to have a “gatekeeping” function against pathogenic bacteria including *S. aureus*, though no mechanistic link has been established (Bomar et al., 2016). Previous research in our department has found that while *Corynebacterium* and *Staphylococcus* species are among the most common organisms isolated from the sinuses of both healthy and CRS patients, CRS patients have a relative reduction in *Corynebacterium* load and expansion of *Staphylococcus* species (Ramezanpour et al., 2018). This study shows that *C. accolens* cell-free culture supernatants have an effect on *S. aureus* growth and cell-free culture supernatants activity *in vitro*.

In this study, we found that *C. accolens* cell-free culture supernatants have a direct effect on the planktonic growth of both the reference and clinical strains of *S. aureus.* This is consistent with Menberu et al. findings, which demonstrated that *C. accolens* has antimicrobial activity against *S. aureus* and MRSA CIs in both planktonic and biofilm forms (Menberu et al., 2021b). This supports the findings of other studies that demonstrate a negative correlation between *S. aureus* abundance and *Corynebacterium* abundance (Uehara et al., 2000; Lina et al., 2003; Frank et al., 2010) and may explain the typical lack of *S. aureus* expansion in the non-diseased state. It also supports the hypothesis of this commensal bacterium’s “gatekeeping” function. The *C. accolens* cell-free culture supernatants had no effect on *S. aureus* biofilm growth, implying that the biofilm structure confers some protection against the *C. accolens* cell-free culture supernatants. Biofilm resistance to naturally and synthetic therapeutic agents has been well documented, which explains their association with the chronic disease state (Dong et al., 2020; Zhang et al., 2020).

Consistent with the reported commensal role of *C. accolens* (Miling et al., 2013; Mahdavinia et al., 2016), we did not observe any detrimental effects of its cell-free culture supernatants on epithelial integrity, membrane permeability, or cellular viability. This was in contrast to the cell-free culture supernatants of *S. aureus* which resulted in marked reductions in TER and increases in FITC-Dextran permeability when applied to HNECs in our study. This aligns with the findings of previous studies that demonstrate a detrimental effect of *S. aureus* exotoxins on sinonasal epithelium (Malik et al., 2015; Murphy et al., 2018b). The intracellular localization of *S. aureus* in CRS patients has been reported and shown to be associated with poor prognostic characteristics and treatment resistance (Tan et al., 2013; Ou et al., 2016, 2017).

The ratio-dependent increase in time to epithelial disruption and membrane permeability when *S. aureus* was co-cultured with *C. accolens* was another finding that supported the proposed protective action of *C. accolens.* The higher the concentration of *C. accolens*, the longer it took for the *S. aureus* cell-free culture supernatants to cause significant damage. We hypothesise that protective factors secreted by *C. accolens* may mitigate *S. aureus-*induced inflammation. This is further supported by the reduction in the release of potent inflammatory cytokines like IL-6 and IL-8 when the cell-free culture supernatants of *S. aureus* co-cultured with the *C. accolens* were applied to primary epithelial cell cultures compared to cell-free culture supernatants of *S. aureus* alone.

Our findings do support the consistent observation of both clinical and the microbiota sinus studies-the association of *S. aureus* with more severe disease and the collapse of the diverse healthy microbiota in patients with CRS (Ramakrishnan et al., 2013; Choi et al., 2014; Kristi et al., 2015; Wagner et al., 2016). Furthermore, our study suggest a mechanistic link by which commensal bacteria like *C. accolens* keep pathogenic bacteria in check through their secreted products. According to a previous study conducted by this group, *C. accolens* cell-free culture supernatants lost their antimicrobial activity against *S. aureus* strains after proteinase K treatment, supporting the hypothesis of the proteinaceous nature of the *C. accolens* secreted products responsible for their anti-staphylococcal effect (Menberu et al., 2021b). However, other secreted products, such as acetic acid and lactic acid or other peptide components, may contribute to these anti-staphylococcal effects. Regardless, a reduction in the number of these gatekeeping bacteria may therefore be implicated as an initiating event in the pathogenesis of CRS.

This study has several imitations which should be noted including its *in vitro* study design, its small sample of clinical isolates tested, and the lack of functional testing. Although IL-6 and IL-8 are excellent surrogate markers for measuring the inflammatory response in CRS patients in the *in vitro* settings, these selected markers can not truly inform us of what its occuring in an *in vivo* setting. Other cytokines, such as IL-1β, CCL-20, CCL-5, CCL-22, IL-33, IL-1α, CCL-2, IP-10, GM-CSFand HBD-2, need to be investigated in the *in vivo* setting and proteomic and molecular work is required to better define the nature of protective factors that are secreted by *C. accolens*. In our study, we mainly tested the mucosal barrier effects of cell-free culture supernatants of *C. accolens* or the cell-free culture supernatants of *C. accolens* and *S. aureus* co-cultures. Considering that the bacterial culture medium may also affect the metabolism of bacteria, further studies are needed to define the exact underlying mechanism and interaction among the live *C. accolens*, *S. aureus* and other potential species that may be present in the upper airway.

## Conclusion

This study demonstrated that *C. accolens* cell-free culture supernatants can inhibit the growth of *S. aureus* planktonic bacteria, and reduce the detrimental effects of *S. aureus* cell-free culture supernatants on the sinonasal epithelium. Further research is required to better characterize the proteins or other peptide components that mediate their effect and their precise mode of action, as well as to evaluate the different inflammatory cytokines and antimicrobial activity between the various species using *in vivo* animal models.

## Data availability statement

The original contributions presented in the study are included in the article/Supplementary material, further inquiries can be directed to the corresponding author.

## Ethics statement

The studies involving human participants were reviewed and approved by The study was approved by the Central Adelaide Local Health Network Human Research Ethics Committee (HREC/15/TQEH/132). The patients/participants provided their written informed consent to participate in this study.

## Author contributions

All authors listed have made a substantial, direct, and intellectual contribution to the work, and approved it for publication.

## Funding

This study was supported by a grant from the National Natural Science Foundation of China (no. 82071023).

## Conflict of interest

The authors declare that the research was conducted in the absence of any commercial or financial relationships that could be construed as a potential conflict of interest.

## Publisher’s note

All claims expressed in this article are solely those of the authors and do not necessarily represent those of their affiliated organizations, or those of the publisher, the editors and the reviewers. Any product that may be evaluated in this article, or claim that may be made by its manufacturer, is not guaranteed or endorsed by the publisher.
