# Corrigendum: *Corynebacterium accolens* inhibits *Staphylococcus aureus* induced mucosal barrier disruption

**DOI:** 10.3389/fmicb.2023.1279422

**Published:** 2023-09-01

**Authors:** Shuman Huang, Karen Hon, Catherine Bennett, Hua Hu, Martha Menberu, Peter-John Wormald, Yulin Zhao, Sarah Vreugde, Sha Liu

**Affiliations:** ^1^Department of Surgery-Otolaryngology Head and Neck Surgery, Basil Hetzel Institute for Translational Health Research, Central Adelaide Local Health Network, Woodville South, SA, Australia; ^2^Adelaide Medical School, The University of Adelaide, Adelaide, SA, Australia; ^3^Department of Rhinology, The ENT Hospital, The First Affiliated Hospital of Zhengzhou University, Zhengzhou, China; ^4^Department of Otolaryngology, Head and Neck Surgery, Shanghai General Hospital, Shanghai Jiaotong University, Shanghai, China

**Keywords:** *Corynebacterium accolens*, *Staphylococcus aureus*, planktonic, biofilm, TER, cell-free culture supernatants

In the published article, there was an error in Figure 4 as published. One of the images (sc+90% C1) was misplaced. The corrected Figure 4 and its caption appear below.

**Figure 4:**
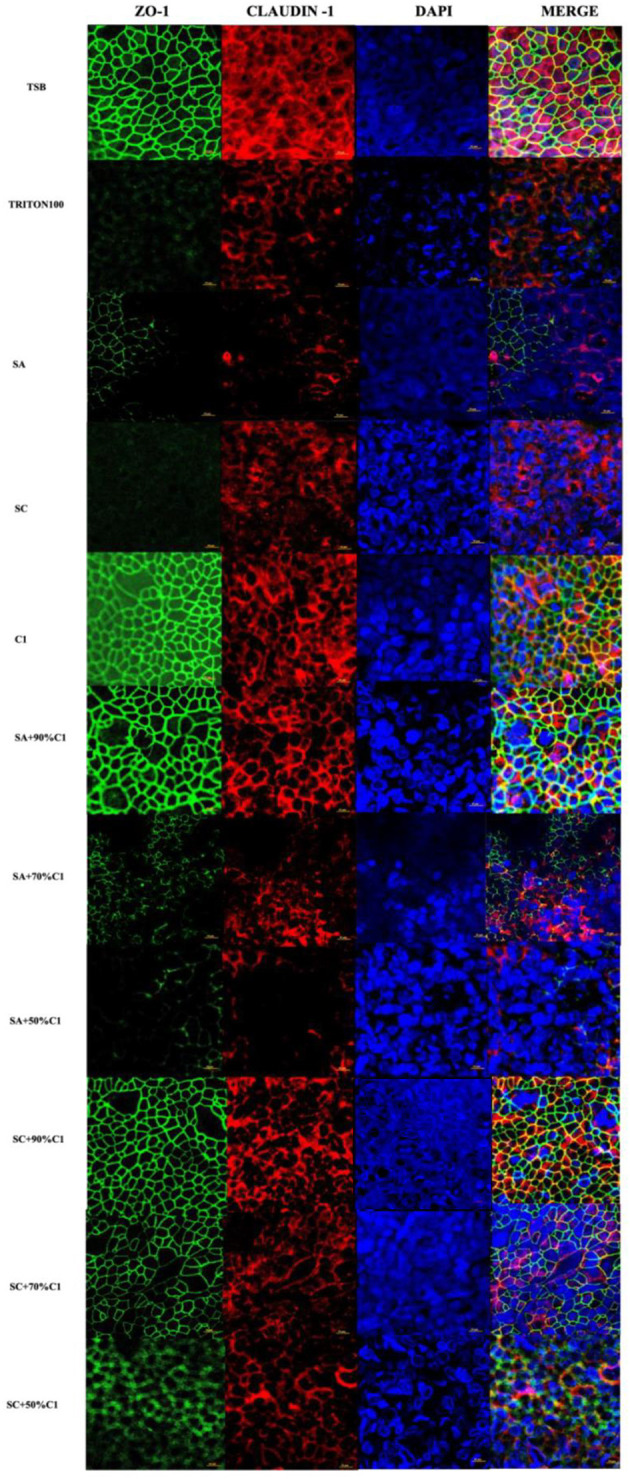
*Corynebacterium accolens* cell-free culture supernatants reduce *S. aureus* cell-free culture supernatants-induced detrimental effects on HNEC-ALI cultures tight junctions. Immunofluorescence staining of tight junction proteins of HNEC-ALI cultures treated with cell-free culture supernatants from SA and SC co-cultured with *C. accolens* in different ratios. HNEC-ALI cultured cells were stained with antibodies against Z0-1(green), claudin-1 (red) and DAPI to resolve nuclei (blue). TSB treatment was used as the negative control. Triton-100 was used as the positive control. Images were examined with confocal laser-scanning microscope (Scale bar = 10 μm). C1, *C. accolens* clinical isolate 1; SA, *S. aureus* ATCC51650; SC, *S. aureus* clinical strain.

The authors apologize for this error and state that this does not change the scientific conclusions of the article in any way. The original article has been updated.

