# Supplementary figures and images for: Corynebacterium accolens inhibits Staphylococcus aureus induced mucosal barrier disruption
Source: Front Microbiol. 2022 Sep 14;13:984741. doi: 10.3389/fmicb.2022.984741 (PMC9515799; doi:10.3389/fmicb.2022.984741)

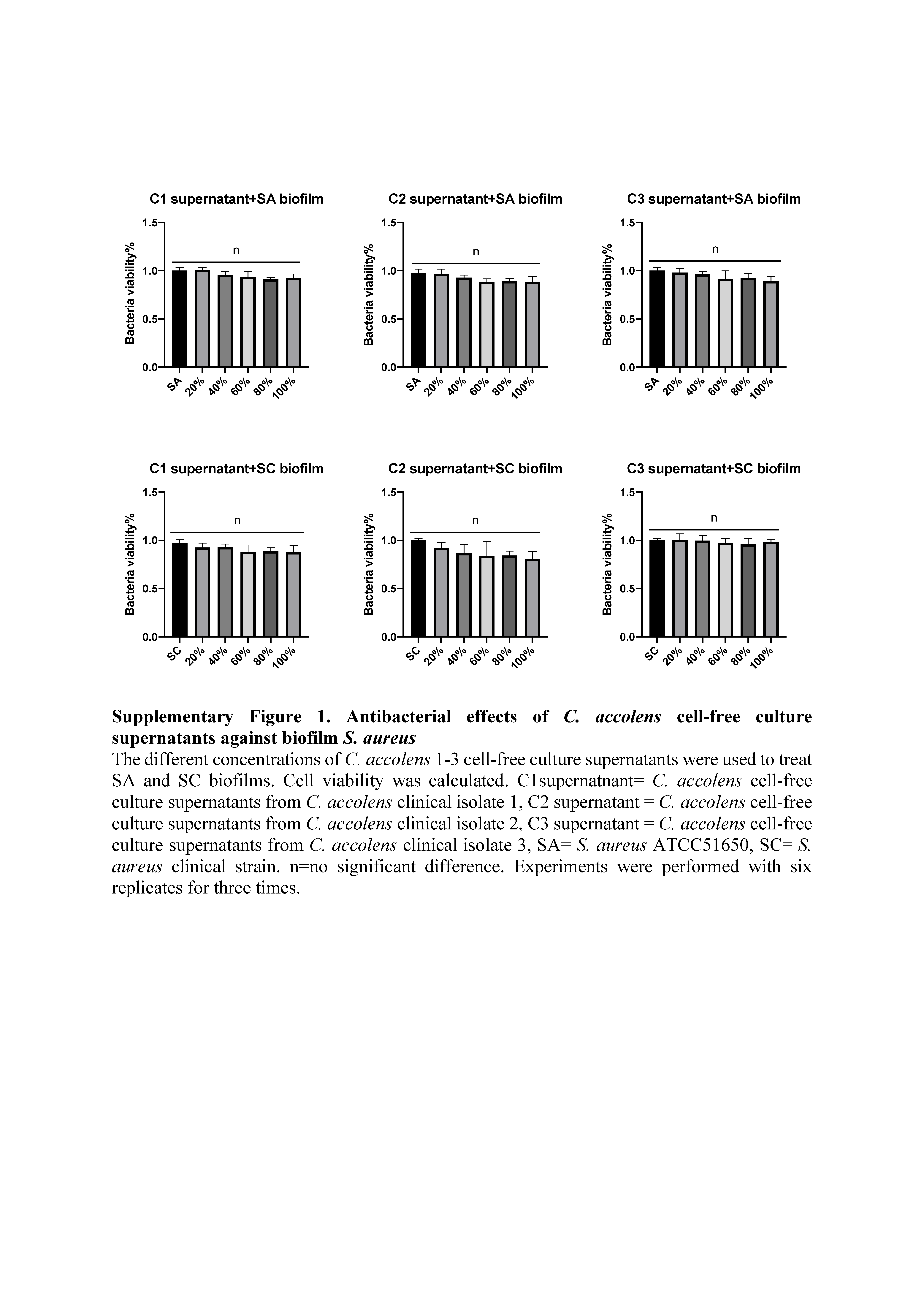

Supplement: Supplementary file 1 [file Image_1.TIFF]

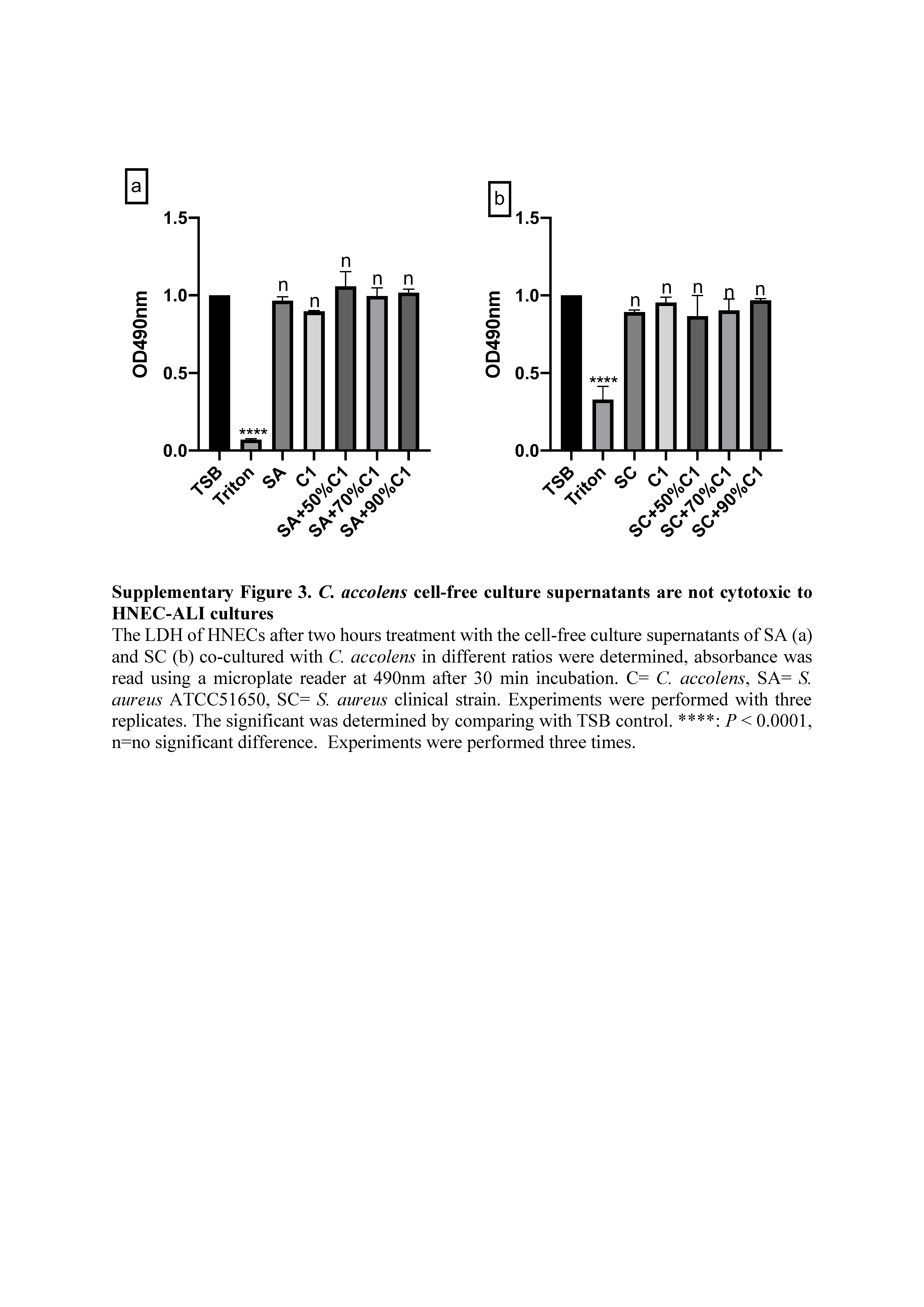

Supplement: Supplementary file 2 [file Image_2.TIFF]

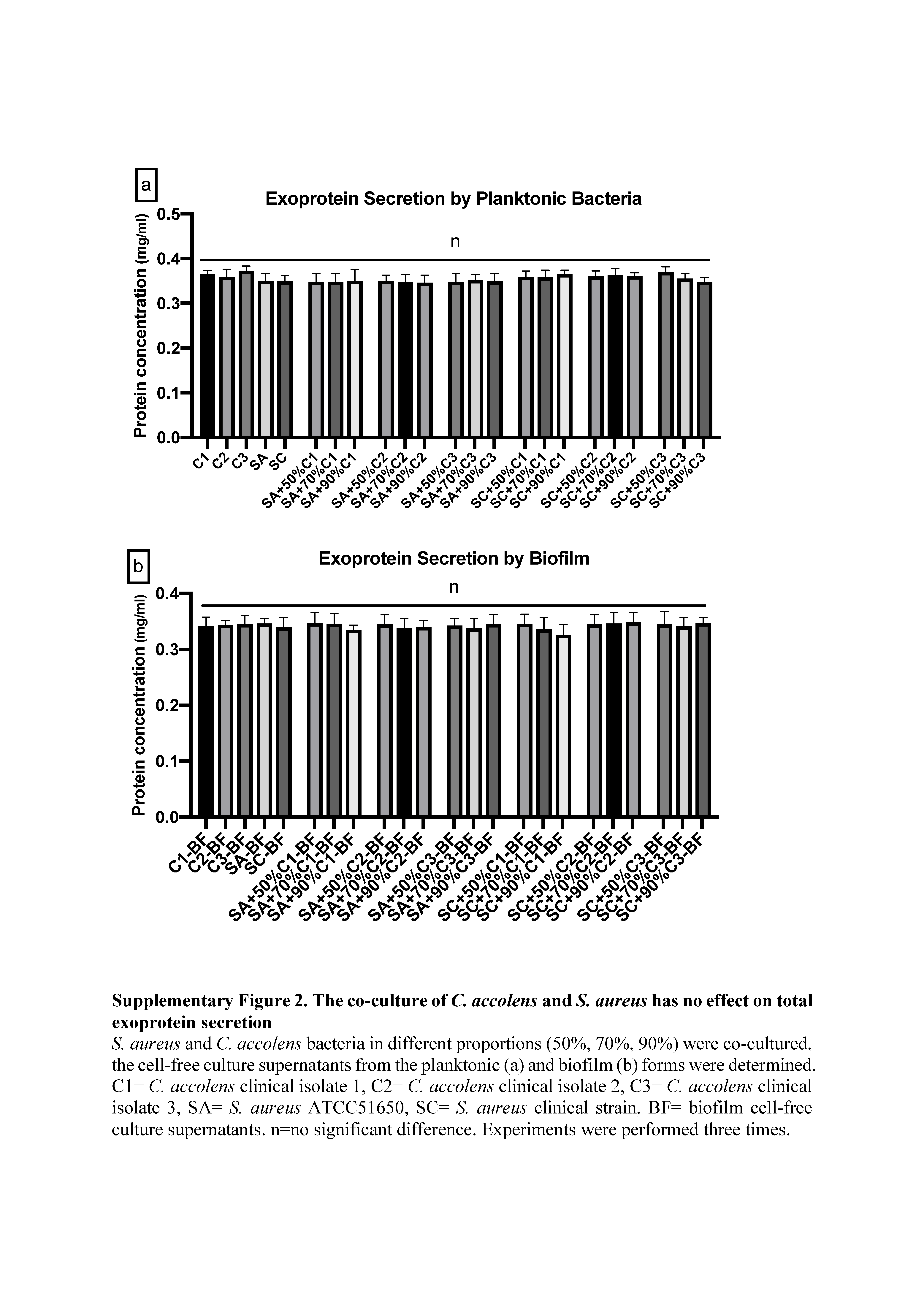

Supplement: Supplementary file 3 [file Image_3.TIFF]
